# Struggling with overweight or obesity in children – fathers’ perceptions and experiences of contributing factors, role and responsibility

**DOI:** 10.1080/17482631.2022.2093912

**Published:** 2022-07-03

**Authors:** Elin Salemonsen, Anne Lise Holm, Kirsten Gudbjørg Øen

**Affiliations:** a Faculty of Health and Social Sciences, Department of Health and Caring Science, Western Norway University of Applied Sciences, Haugesund, Norway; b Faculty of Health Sciences, Department of Public Health, University of Stavanger, Stavanger, Norway

**Keywords:** Childhood overweight and obesity, contributing factors, family functioning, fatherhood, responsibility, role

## Abstract

**Purpose:**

The family environment is crucial in the prevention and treatment of childhood obesity; however, there is a lack of knowledge concerning paternal perceptions and experiences in childhood weight management. The **aim** of this study was to explore (i) perceptions of contributing factors to childhood overweight and obesity among fathers of children with overweight or obesity and (ii) the fathers’ experiences of their parental role caring for a child with overweight or obesity.

**Method:**

A qualitative content analysis was conducted of data from semi-structured in-depth interviews with eight Norwegian fathers of ten children and adolescents with overweight or obesity.

**Results:**

The analysis identified one overall theme; *Feeling uncertain and struggling to understand their own responsibility for the child’s overweight or obesity*, which consists of two themes; 1)*Trying hard to figure out the child’s obesity as a complex interaction of factors* and 2)*Family functioning—negotiating roles and responsibility in parenthood.*

**Conclusion:**

Fathers must be met with understanding about their uncertainty and their struggle to understand their individual responsibility for their child’s weight excess. It can be necessary to address the significance of family functioning, collaboration, responsibility within the familiy in addition to structural responsibility in clinical dialogues and counselling in order to help with weight management

## 1. Introduction

Worldwide, the prevalence of overweight (ISO-BMI ≥ 25) or obesity (ISO-BMI ≥ 30) in children under the age of 5 was 38 million in 2019, and over 340 million children and adolescents aged 5–19 had overweight or obesity in 2016 (World Health Organization, 2021). The rising BMI in children and adolescents continues in low- and middle- income countries and have accelerated in parts of Asia. However, the rising BMIs have plateaued in many high-income countries, even though at high levels (Abarca-Gomez et al., 2017). A total of 15–20% of children and 25% of adolescents in Norway have overweight or obesity (The Norwegian Institute of Public Health, 2017). Children with obesity tend to remain obese in adulthood (World Health Organization, 2016).

Childhood obesity affects both physical and psychosocial health and is associated with a higher risk of poorer health, disability in adulthood and premature death (World Health Organization, 2021). Children with obesity are at high risk of multiple comorbidities (Kumar & Kelly, 2017) and are at an increased risk of fractures, breathing difficulties, hypertension and insulin resistance (World Health Organization, 2021). In addition, obesity can affect a child’s educational attainment and quality of life. Children and adolescents with obesity experience weight stigma (Pont et al., 2017), bullying and fragile social relationships (Øen et al., 2018), and are at an increased risk of poorer psychosocial functioning (Halfon et al., 2013). Halfon et al. found an association between obesity and mental health in children aged 10–17 (Halfon et al., 2013). Weight-related stigmatization also has an impact on medical treatment in primary care (Schwenke et al., 2020).

Obesity is due to a complex interaction between biological, social, developmental, behavioural and environmental factors (The Obesity Society, 2021; Yumuk et al., 2015). There is consensus that the fundamental cause of overweight and obesity is an energy imbalance between calories consumed and calories expended (World Health Organization, 2021). This energy imbalance partly stems from changes in the marketing, affordability, availability and types of food (World Health Organization, 2016). Genetic and environmental factors have a strong effect on BMI (Silventoinen et al., 2010), and many children today are growing up in an obesogenic environment (World Health Organization, 2016). Increasing urbanization and technology developments have led to greater physical inactivity, with more time being spent on screen-based and sedentary leisure activities (World Health Organization, 2016). In addition, energy-dense food, which is high in fat and sugars, is easily accessible. Both environmental and societal factors have an impact on the development of overweight and obesity (The Obesity Society, 2021; World Health Organization, 2021; World Health Organization, 2016). This also includes family environment (family functioning) and parenthood (East et al., 2019; Eg et al., 2017; Halliday et al., 2014; Mazzeschi et al., 2013; Sigman-Grant et al., 2015). The Family Ecological model (Davison et al., 2013) is useful to illustrate and understand contextual and family systems factors affecting parenting specific to healthy lifestyle.

Parents model eating behaviours and help to shape children’s food intake (Birch & Davison, 2001; Birch & Fisher, 1998; Savage et al., 2007), and how a child’s eating and activity habits develop is influenced by both parents (Davison et al., 2018). Fathers are more actively engaged in meal preparation and feeding their child in contemporary families and changing family structures (Jansen et al., 2018). A qualitative study in which 37 fathers were interviewed revealed that 62% of the fathers shared food parenting responsibility with their child’s mother (Khandpur et al., 2016). Fathers’ food parenting style was a predictor of their children’s eating behaviour, and this review indicates that fathers play a key role in influencing children’s eating behaviour (Litchford et al., 2020). We know that family-based interventions are effective (Chai et al., 2019; Kumar & Kelly, 2017; Ranucci et al., 2017), and family interventions should include both parents (Davison et al., 2018). In recent decades, fathers have become more involved in childhood development and healthcare, including encouraging healthy eating and exercise, and monitoring their child’s well-being and development (Garfield & Isacco, 2012). Men’s involvement in childcare has become an accepted component of modern masculinity (Haavind, 2011, 2006). However, the changing role of fathers in childhood obesity is understudied (Jansen et al., 2018; Morgan et al., 2017; Wong et al., 2017).

Literature and studies on parental roles in children’s and adolescent’s lifestyles typically equate the term “parent” with “mother” and mothers as primary caregivers. Davison et al. found fathers represented only 6% of all parent participants in family-based interventions targeting childhood obesity prevention (Davison et al., 2018). A study by Morgan et al. concluded that existing paediatric obesity treatment or prevention programmes with parent involvement have not engaged fathers (Morgan et al., 2017). Recruitment and engagement of fathers in obesity research need to be tailored (Haavind, 2011) in the same way as strategies aimed at other difficult to reach populations, as fathers are a valued and important part of a child’s life, and failure to include them may represent “poor research practice”.

Previous quantitative cohort studies revealed an association between fathers’ involvement in caregiving and childhood overweight and obesity, and that greater involvement by fathers may help reduce the risk of obesity among young children (Sato et al., 2020; Wong et al., 2017). A literature review by Neshteruk et al. showed a modest association between father and child physical activity, and that additional research is needed in order to better understand the specific factors that influence this relationship (Neshteruk et al., 2017). One qualitative study calls for policymakers and public health practitioners to consider the contribution of fathers and to work to include fathers in diet-related efforts (Fielding-Singh, 2017). A qualitative study by Anti et al. from the perspective of healthcare practitioners found that fathers have a minimal role in the management of their child’s overweight and obesity, which may lead them to neglect the father’s role with regards to this important issue (Anti et al., 2016).

The Norwegian Directorate of Health, (2010) recommend that general practitioners (GPs) and public health nurses (PHNs) in child health clinics and school health services in primary care act at both individual and structural level to prevent the development of overweight and help prevent and reduce obesity among children and adolescents. We recognize that both parents are critical stakeholders in childhood overweight and obesity prevention. As the literature review in this introduction reveals, there is a particular need to recruit and engage fathers of children and adolescents in prevention efforts. No studies have been found that explore what Norwegian fathers perceive to be contributing factors to childhood obesity or that provide descriptions of their experiences in their parental role in weight management. Knowledge about fathers’ understanding of childhood overweight or obesity as well as their parental role could be helpful for healthcare professionals and PHNs when they provide help and support to families, and to meet the fathers’ needs in overweight and obesity management. In this study, we respond to calls for more research on the father’s perspective. Thus, the aim of this study was to explore (i) perceptions of contributing factors to childhood overweight and obesity among fathers of children with overweight or obesity and (ii) the fathers’ experiences of their parental role caring for a child with overweight or obesity, with the intention of informing health professionals how to meet the fathers’ needs in overweight and obesity management.

## 2. Materials and methods

An interpretative explorative design (Polit & Beck, 2017) was used to increase the understanding of contributing factors of childhood overweight or obesity and the parental role from the father’s perspective. An explorative design was chosen due to the sparse knowledge on fathers’ experiences and perception of childhood weight management. A qualitative approach can help discover themes or patterns based on perceptions and experiences to understand behaviour in order to inform clinical practice (Polit & Beck, 2017).

### 2.1 Setting

In Norway, child health clinics and school health services in primary care offer help, advice and support from PHNs, GPs and physiotherapists to children and adolescents (age 0–20 years) and their parents (Helsenorge, 2022). Nearly 100% of children and their family uses these free of charge services and follow up the consultations offered. PHNs in these child health services act at both structural and individual level to prevent the development of overweight and help prevent and reduce obesity among children and adolescents (The Norwegian Directorate of Health, 2010). In Norway, a total of 15–20% of children and 25% of adolescents have overweight or obesity (The Norwegian Institute of Public Health, 2017). PHNs are measuring children’s height and weight at given consultations and are in first line to follow up those children and adolescents identified having overweight or obesity. Children, adolescents and their parents are free to contact these child health services at any time if they need help and support to manage the child’s weight excess.

### 2.2 Participants and recruitment

PHNs working in four municipalities, including nine child health clinics and 15 school health services in Western Norway were asked to recruit fathers from families where the family (either father, mother or both) were followed up by PHNs due to the child’s weight condition, or had contacted the local child health services to obtain help for their children’s weight excess. Some of the fathers were recruited by the PHN through the child’s mother, and some of them in person, by telephone or in counselling. The inclusion criteria were fathers who had custody to their child, had children or adolescents who had overweight or obesity (ISO-BMI > 25), and lived with their child part or fulltime. The participants in our study were recruited from three local child health clinics and two school health services.

In this study we asked for the fathers’ perceptions of contributing factors to their children’s overweight or obesity and their experiences of their parental role in caring for a child with overweight or obesity. Purposive sampling (Malterud et al., 2016) was therefore used to find fathers with experience from their care of children with overweight or obesity. A total of eight fathers from one small (rural) and two medium-sized (urban) municipalities in Western Norway volunteered to participate in the study. They were all involved in the family’s efforts to manage their child’s weight excess. The families have had contact with PHNs in child health services about this condition between three months to over a year. This included 10 children, aged 4–16 years (Table I).

**Table I.** Participant characteristics.

Characteristics	Number of participants
**Gender:**	
Male	8
**Age:**	34–55
Mean age	45
**Civil status:**	
Single/divorced	1
Partner/married	7
**Education:**	
Secondary school	6
Bachelor’s degree or higher	2
**Occupational status:**	
Employee 100%	8
Daytime	4
Shift-work	4
**Habitat:**	
Rural	3
Urban	5
**Children/adolescents:**	
Age	4–16

### 2.3 Data collection

A thematic interview guide with open-ended questions was developed by first author in line with Kvale and Brinckmann (Kvale & Brinkmann, 2015) (Table II). First author contacted all the fathers who consented to participate, and conducted eight individual in-depth interviews with the fathers in Norwegian. In accordance with the participants’ wishes, all the interviews took place in their local child health clinic. The participants were informed of the purpose of the study, and confidentiality was emphasized. The form of the interviews was open, which allowed for elaboration, and lasted between 80 and 90 minutes. Audio recordings were made of the interviews and these were transcribed verbatim by the first author. The empirical data provided rich and detailed descriptions of experiences from the participants perspective. The quality of data, study design, the narrow aim and the nature of the topic, like access to participants who wanted to talk about this sensitive topic, as “hard to reach population”, guided the sample size (Malterud et al., 2016; Morse, 2000; Sandelowski, 1995).

**Table II.** Thematic guide for individual interviews.

**Understanding of overweight and obesity in children** How do you prefer to talk about your child’s overweight? What is your understanding/experiences of your child’s overweight? What do you perceive or experience as possible causes of overweight or obesity in your child?
**Parental role in prevention and treatment of overweight and obesity** What do you think can help prevent or reduce overweight or obesity? How do you see your responsibility? What are your expectations for yourself and the child’s mother? How is your family’s division of labour and responsibility? What is important for you to prioritise?

### 2.4 Data analysis

Data were analysed in line with the analytical steps in Qualitative Content Analysis suggested by Graneheim & Lundman (Graneheim & Lundman, 2004) and Graneheim, Lindgren & Lundman (Graneheim et al., 2017). First author was responsible for the analysis and received input from the co-authors. The authors tried to understand the meaning of the text in the light of their own experiences as PHNs (first- and third author) and mental health nurses (second- and third author), and now as researchers. Such pre-understanding may have influenced the interpretation of the analyses (Denzin & Lincoln, 2012).

The first step included reading the interviews open-mindedly to gain an impression of the text as a whole and an understanding of each interview. This first reading revealed several aspects of fathers’ perceptions and experiences, which were presented as meaning units and categories (Tables IV and V). In the second step, the meaning units were sorted into two areas. In the third step, the text was condensed as outlined by Graneheim and Lundman (Graneheim & Lundman, 2004). The number of words was reduced, while preserving the core content. The fourth step involved abstraction by means of reading and comparing. The abstractions were related to similar content and grouped together. During this analytic process, the authors met several times to discuss the emerging themes and sub-themes in order to interpret and remain close to the text (Table III). The authors agreed to interpret the text into one overall theme comprising two themes and six sub-themes.

**Table III.** Overall theme, themes and subthemes describing fathers’ perceptions of contributing factors to childhood obesity and their view of their parenting role.

Overall theme: *Feeling uncertain and struggling to understand their own responsibility for the child’s overweight or obesity*	
Subtheme	Theme
Biological factors	Trying hard to figure out the child’s obesity as a complex interaction of factors
Psychosocial factors	
Societal development and structural factors	
Recognizing the child’s weight excess	Family functioning—negotiating roles and responsibility in parenthood
Responsibility and prevention strategies within the family	
Family dynamics and climate

**Table IV.** Example of qualitative content analysis theme 1.

Meaning unit/Category	Subtheme	Theme	Overall theme
*Consumption and availability of energy-dense food*	Societal development and structural factors	Trying hard to figure out the child’s obesity as a complex interaction of factors	*Feeling uncertain and struggling to understand their own responsibility for the child’s overweight or obesity*
*Advertising, pricing policy*			
*Lack of cycle and walking paths*			
*Technology development and societal development*			
*Time pressure*			
*Governmental responsibility and priorities*			
*Political responsibility*

**Table V.** Example of qualitative content analysis theme 2.

Meaning unit/Category	Subtheme	Theme	Overall theme
*Agreements and disagreements about limits*	Family dynamics and climate	Family functioning—negotiating roles and responsibility in parenthood	*Feeling uncertain and struggling to understand their own responsibility for the child’s overweight or obesity*
*Family climate and interaction*			
*Dialogue and cooperation*			
*Building good relations with the child and within the family*			
*Division of labour*			
*Priorities*			

### 2.5 Trustworthiness

As qualitative content analysis is used in this study, it is important to establish trustworthiness (Kyngäs et al., 2020; Lincoln & Guba, 1985) by using the concepts *confirmability, credibility, dependability and transferability. Confirmability* is a measure of how well the study findings are supported by the collected data (Kyngäs et al., 2020; Lincoln & Guba, 1985). This aspect of trustworthiness is concerned with the connection between the data and the results. Hence, when considering confirmability, the authors of this study are aware that the findings could have been shaped by the data collection. In addition, we are aware that the data could be a result of our own bias, interests and attitudes (Kyngäs et al., 2020; Lincoln & Guba, 1985). Confirmability was strengthened by discussing the themes, sub- themes and overall theme between the authors on several occasions to find the most appropriate interpretation. *Credibility* is concerned with whether or not the research findings seem to represent a credible interpretation of the original data (Lincoln & Guba, 1985). Direct quotation from the participants perspective demonstrated credibility in the interpretation. *Dependability* is defined as an assessment of the quality of the integrated research processes. It refers to the stability of data over time and varying conditions and the potential for replication (Lincoln & Guba, 1985). As authors, we have demonstrated dependability through description of the research process from the start, including context, participants, data collection and analysis. *Transferability* describes the degree to which research findings can be applied to other fields and contexts (Lincoln & Guba, 1985). According to Kyngäs et al. (Kyngäs et al., 2020), researchers should be concerned about transferability and question whether their results will hold in another settings or for other groups of participants. It is important to note that transferability is not the same as generalization in quantitative research because transferability is also concerned with how readers will extend the results to their own situations, whereas generalization covers the extension of results from a sample to a broader population (Kyngäs et al., 2020). Therefore, the setting and participants have been thoroughly described in our study.

### 2.6 Ethical issues

This study was registered and approved at the Norwegian Centre for Research Data (NSD), project number 35008, and the ethical guidelines in the Helsinki Declaration were followed. Informed consent for study participation was emphasized and each participant gave their written consent to participate prior to the interviews. The participants received both an oral and a written invitation and information about the study. Participation in the study was voluntary and the participants were informed about their right to withdraw at any time. Before the individual interviews were held, precautions were taken by reflecting on how to take care of the participants if the interview situation became unpleasant or challenging. Parents of children with excess weight are often blamed and shamed for their children’s weight (Gorlick et al., 2021). We were therefore aware of the potential reactions of the fathers when discussing this sensitive theme. The interview setting was well prepared, and the emphasis was on creating a respectful and non-judgemental atmosphere.

## 3. Results

The analysis identified one overall theme; *Feeling uncertain and struggling to understand their own responsibility for the child’s overweight or obesity*, which consists of two themes; 1) *Trying hard to figure out the child’s obesity as a complex interaction of factors* and 2) *Family functioning—negotiating roles and responsibility in parenthood*. Each theme comprised several sub-themes (Table III).

### 3.1 Feeling uncertain and struggling to understand their own responsibility for the child’s overweight or obesity

The overall theme *Feeling uncertain and struggling to understand their own responsibility for the child’s overweight or obesity* reflected the fathers’ perceptions of contributing factors to childhood overweight and obesity and the fathers’ experiences of their parental role caring for a child with overweight or obesity. The fathers felt uncertain about the exact reason for their child’s overweight, how they could help their children and a frustration about not always being able to help. They felt responsible for their child’s diet and physical activity (PA), but also recognized that childhood obesity includes other biological factors like genes and sleep, psychosocial- and, environmental factors, including family dynamics and climate, and structural factors like policy and societal development. This complexity made it even more difficult for the fathers to understand how to help. This overall theme seems to be a red thread throughout the data, themes and subthemes. We will elaborate further on the findings below.

#### 3.1.1 Trying hard to figure out the child’s obesity as a complex interaction of factors

The first theme described the fathers’ knowledge about contributing factors to overweight and obesity in children and how they are trying hard to figure out their child’s obesity as a complex interaction between biological, psychosocial and structural factors, as well as questions concerning individual and structural responsibility.

*Biological factors*

All the fathers perceived there to be an imbalance between the diet and the activity level which leads to overweight, and believed it was important to find the right balance between an adequate amount of healthy food and physical activity in order to prevent overweight and obesity. However, they consider the picture to be complex, and believe there are several contributing and interacting factors. The fathers had different opinions and experiences of the causes of their child’s overweight. Four of the fathers said that their children are physically active and believe that the issue is solely about the diet; overeating caloric food containing high amounts of sugar and fat. Most of them felt that their child or adolescent spends too long on sedentary activities, like watching TV, sitting at the computer, using their Play Station, gaming and homework, and that there is too little time for playing and physical activity. They described how their children have adopted new ways of socializing through the use of various social media platforms, and some of the fathers expressed a concern for their child’s movement development. One of the fathers expressed the following:

*In today’s society, children are not out playing. They sit in their rooms, logged on to their computer or some other device, and have contact with their friends there. I find it frustrating. It’s frustrating to see all the time he spends on the internet and gaming. It’s like an addiction. (Participant 2)*

Another father believed that lack of sleep is a contributing cause and described the differences between his child with overweight and his normal-weight child when it comes to sleep duration. One of the fathers believed that it has to do with differences in calorie expenditure. Several of the fathers were certain that inheritance and genes play an important part in their child’s overweight.

*In nature, those equipped with the best genes will survive. Those who were able to gain weight and stay healthy won the battle … . However, in contemporary society with the abundance of food, we no longer need these genes. At one point they became too much. (Participant 8)*

*Psychosocial factors*

The fathers believed that how their child feels about his/herself (body and mind), their well-being and if and how they receive social and psychological support will impact on their way of living and management of life. One of the fathers described how his son started to gain weight when the family moved to another city, and he had problems finding new friends. Moving to another residence led to isolation, even more sedentary activity and emotional eating. One of the other fathers believed there was a link between his son getting bullied, isolation and emotional eating. Several of the fathers believed that major disagreements between parents about how to raise their child, setting boundaries and conflicts between parents can cause overweight in children. One of the fathers expressed the following:

*I believe that it is very important for parents to cooperate properly and that happy parents lead to happy children. If my child’s mother and I have argued, the percentile would most certainly turn even more upwards. (Participant 4)*

*Societal development and structural factors*

All the fathers cite the societal development in the last three decades as a possible contribution to overweight and obesity. Most of the fathers highlight social changes, where both parents are working full-time and experiencing time pressure, which makes it easy to resort to simple solutions and fast food.

*We live in a society where everything is on fast forward, where both parents are working full-time and are late home. This makes it easier to sometimes buy fastfood on your way home. We pay others to make our meals, no wonder we are gaining weight*. (Participant 2)

The fathers expressed concern regarding the technological developments in media, mobile devices and data. Several of the fathers regarded this development as positive, however, they believe there is a flip side to the coin. The variety of screen-based activities is extensive and leads to a sedentary lifestyle.

All the fathers have concerns about the abundance, variety and availability of unhealthy food, and consider this to be one of the major causes of the increase in overweight and obesity in today’s society. The fathers believe that today’s society is mostly focused on consumption and growth. They experience major pressure to buy through different media, and in grocery stores they feel pressured to buy more than they need. The fathers feel that they are constantly exposed to marketing and advertising and find it worrisome that unhealthy food and beverages are so cheap.

*I sometimes get so frustrated in the grocery store when the adverts say ‘3 for the price of 2’. I just want to buy one*. (Participant 7).

Several of the fathers believe that it is politicians’ and the government’s responsibility to facilitate healthier choices, through pricing policy, such that healthy food becomes cheaper, i.e., reducing taxes on fruit and vegetable. They would also like to see less advertising in grocery stores targeting children and sweets being placed out of the reach of small children—away from the checkout.

The fathers living in urban areas find that local governments do facilitate cycling and walking, and that there are various recreation areas and sport halls they can use nearby. All the fathers living in rural areas believe that the local authority is not making the right priorities in relation to prevention and adaptation in the local community. This is exemplified by the lack of responsibility for and prioritization and construction of cycle and walking paths that would enable children to walk or cycle to school.

*I believe that the local politicians have their priorities all wrong when it comes to cycle and walking paths. We have tried for several years to make them see the importance of these*. (Participant 2)

#### 3.1.2 Family functioning—negotiating roles and responsibility in parenthood

The second theme described the fathers’ perceived parental role caring for a child with overweight or obesity in terms of family functioning and negotiation of roles and responsibility.

*Recognizing the child’s weight excess*

All the fathers in this study recognize that their child is overweight and want to do something to help them. The fathers believe it is important to be aware of whether their child is putting on weight. They did not like using the words “overweight” or “obese”, and preferred instead descriptions like “big”, “round”, “being in good condition”, “having to watch out for” or “tendency to put on weight”. One of the fathers expressed the following:

*She was quite big when she was born and has been that way ever since. I do not thinkshe is that big. I tell her she looks nice the way she is. (Participant 8)*

The most important issue for the fathers was that their children followed their own percentile and did not gain more weight. They were trying to focus on their child eating a healthy diet and staying physically active rather than on the excess weight itself. They believed it was important to be open about the issue and that both parents need to be open to recognizing their child’s weight problem and agreeing to address the problem. Some of them experienced parental disagreement concerning the right course of action to help the child. They point to the necessity to realize that excess weight is a problem for the child and to take the matter seriously.

*Responsibility and prevention strategies within the family*

All the fathers expressed that it is first and foremost the parents’ responsibility to prevent overweight and obesity in their children. One of them expressed the following:

*It must be us as parents that have the responsibility. I can’t say anything else. We can’t expect society or someone else to take care of our child in this way*. (Participant 8)

They emphasized the necessity of building a trusting relationship with their child and this was described as a prerequisite for being in a position to help. The fathers highlighted how being available and present and making the child feel safe were important in building trust. They all mentioned engagement, participation and following up their children at home, at school, with homework and in leisure activities. They considered fatherhood to be just as important as motherhood, and believe that fathers and mothers have equal responsibility and that both parents must contribute.

I *see myself as an equal and integral part of parental responsibility and the distribution of tasks. I think it’s important to be a good role model for my kids. It’s good that they see that I’m just as happy to cook as their mother. (Participant 1)*

The fathers believed it is important for them to eat healthy and be active, and that it is a parental responsibility to motivate, be involved, participate and serve as a good role model. One of the fathers highlighted how the family lifestyle and activity level will affect the children’s choice of lifestyle in later life. The fathers described how activity and eating habits are formed within the family.

*What you bring along from your childhood forms the foundation for your own family. I try to learn from my experiences in life*. (Participant 5)

Several of them described having an important task to pass on knowledge to future generations e.g., sweets are for Saturdays. One of the fathers told how this transfer of knowledge and habits happens quite automatically, and that established patterns and habits are followed if we don’t know a better way to do it. Several of the fathers believe that it is important to teach their children healthy habits while they are living at home and to involve them in shopping and cooking. One of the fathers expressed the following:

*Once my son moves out, I will no longer have any control over what he eats*.(Participant 3).

The fathers see the necessity of giving the children knowledge about healthy eating and physical activity. However, they believe that schools and perhaps PHNs at schools should also provide this information. The fathers believed that this information should be repeated throughout the child’s life and in different years at school. Two of the fathers highlighted the necessity of having a PHN to discuss things with their child when they did not meet around the dinner table as a family and have time to talk to each other like before. Other fathers believe that this responsibility lies with them and that they do take responsibility, however, they find it necessary to consult with experts.

Two of the fathers believed that in the case of divorced parents, single parents tend to focus on doing nice things when the child was with them, including exciting activities and enjoying extra good food. Some of the fathers’ described how the child’s mother was stricter and that they as fathers are more permissive. Several of the fathers believe that parents must be better at setting boundaries and at agreeing and cooperating with plans.

*Parents need to be more foresighted and proactive when they see a negative development. I believe the most important thing is to set boundaries for sedentary activities, including reading, homework and screen-based activities. It’s very difficult and can be disregarded very quickly*. (Participant 3)

*Family dynamics and climate*

Several of the fathers recognized that the family function affected how they cared for children with overweight or obesity. This included the family dynamics, relationship, interaction, cooperation, division of labour and dialogue. All the fathers highlighted the importance of good parenting and cooperation for successful childhood weight management. They believe it is important for parents to be able to talk together and be open, and for them to agree on how to raise their children and set boundaries. Dialogue and low levels of conflict between the parents are described as essential by several of the fathers. Some of the fathers believe it is important to “choose your battles” in order to avoid conflicts and bad moods. This applies both to the relationships between the parents and the parent-child relationship. One of the fathers said he found disagreements in parenting style very difficult. Several of the fathers have found that it is difficult to help their overweight child when they as parents disagree, and one of them expressed the following:

*The most important thing is that parents cooperate and agree. It is also important to be flexible, be open about expectations and share the tasks. If we have agreed on a plan, it’s important that we stick to it*. (Participant 6)

Some of the fathers believed that their partner may feel that they as fathers should be more involved, however, most of the fathers in this study said they participated in shopping, cooking, laundry and housekeeping.

## 4. Discussion

The aim of this study was to explore (i) perceptions of contributing factors to childhood obesity among fathers of children with overweight or obesity and (ii) the fathers’ experiences of their parental role caring for a child with overweight or obesity. The overall findings show that the fathers in this study exhibited a high degree of self-reflection and had considered contributing factors to childhood obesity. However, they are uncertain and struggle to understand their own responsibility for the child’s overweight or obesity. The fathers are trying hard to figure out the child’s obesity as a complex interaction of factors, and their experiences in the parental role were reflected in the family functioning and the negotiation of roles and responsibility. This discussion will focus on the overall theme and the two themes explored, and will be discussed in light of previous studies and literature.

The fathers are *feeling uncertain and are struggling to understand their own responsibility for the child’s overweight or obesity*. The fathers feel responsible for their child’s diet and PA, at the same time they see that childhood obesity also includes biological factors like genes, diet and PA, environmental and psychosocial factors, and policy and structural factors. This makes it even more difficult for them to understand and to be able to help. The eternal question of “nature or nurture” comes into play, and the fathers seem to want to discuss responsibility at several levels, including the question of individual and structural responsibility. The need to discuss responsibility may be a response to a sense of guilt or shame for having a child with overweight or obesity. Parents are largely blamed for childhood obesity, and much of the responsibility for reducing childhood obesity is placed on the parents (Wolfson et al., 2015). According to Holm (Holm, 2008), a parent can be seen as causally and morally responsible for their child because their feeding style is a factor in the causal complexity leading to obesity. The parent may not be to blame if this consequence of the feeding style was not predictable or if changing the feeding style is practically impossible (Holm, 2008). Parents of children with excess weight are often blamed and shamed for their children’s weight and mothers of children who are overweight or obese are frequently the target of weight stigma and experience negative cognition and emotions due to their perceived role in their children’s weight (Gorlick et al., 2021). Based on our results of the fathers’ uncertainty, perceived role and responsibility, we suggest that this applies equally to fathers.

The fathers are *trying hard to figure out the child’s obesity as a complex interaction of factors*. The biological and psychosocial factors described by the fathers in our study are in line with the research literature on the causes of overweight and obesity (World Health Organization, 2021). Genetics, food quality and quantity, a sedentary lifestyle or lack of PA are described earlier (Han et al., 2010; World Health Organization, 2021). Parenthood is described as a contributing factor (Huffman et al., 2010) as parents model eating behaviours and help shape children’s food intake (Birch & Davison, 2001; Birch & Fisher, 1998), like the Family Ecological Model (FEM) illustrates (Davison et al., 2013). Behavioural phenotypes for childhood obesity, like individual differences in appetitive traits and genetic predispositions, are described by Kral et al. (Kral et al., 2018), and are in line with these fathers’ understanding of contributing factors to their child’s overweight or obesity. The fathers understand psychosocial factors both as a consequence and a cause of obesity, and this corresponds to the findings on consequences in Halfon et al. (Halfon et al., 2013). Psychological correlates of childhood obesity, such as behavioural and emotional problems, also seem to be two-fold (Puder & Munsch, 2010). Some of the fathers described sleep duration as one possible relationship to the child’s overweight. Sleep duration seems to have an impact on weight gain and is also a risk factor (Börnhorst et al., 2012; Felső et al., 2017). Later sleep timing was related to obesogenic behaviours and may represent an obesity risk factor (Skjåkødegård et al., 2021). This is a factor that parents cannot influence in the same way as offering the child healthy food. Systematic reviews of effects of interventions including diet and/or PA have moderate certainty evidence (Brown et al., 2019), and this substantiates the fathers’ experiences of uncertainty and their struggle to understand how they can prevent overweight or obesity in their children, and also to understand the limits of their responsibility.

The fathers in our study described structural factors and underlined the need for local measures like pavements, cycle and walking paths and recreation areas nearby. Previous studies show that greenspaces and the condition of a neighbourhood have an impact on childhood overweight (Schalkwijk et al., 2018) and that government prioritization of neighbourhood surroundings affect the child and the families’ resources in their fight against overweight or obesity (Wei et al., 2021). According to the WHO’s Ending Childhood Obesity (ECHO) report (World Health Organization, 2016), increased political commitment and responsibility is needed to tackle the global challenge of childhood overweight and obesity. However, political action lags behind the need for governments to recognize their moral responsibility to reduce the risk of childhood obesity (Bauman et al., 2019). In order to improve population health, there is a need for health equity and a wide range of measures that address the environmental causes of overweight and obesity. A whole-of-government approach is also needed, including policies across all sectors that systematically take health into account (World Health Organization, 2016).

Our findings show that the fathers emphasized the structural responsibility, including availability, pricing policy and marketing of unhealthy food. Increased public awareness about overweight and obesity in children may lead to changes in nutrition and activity that are sufficient to curb the rise in mean BMI in children and adolescents (Abarca-Gómez et al., 2017). WHO recommends other price policies, such as lower taxes on healthy food, with a view to changing eating and drinking behaviour (Soares, 2016). Several countries in Europe support a stronger emphasis on industry regulations and the use of taxes, and many countries have taxed sweets and beverages for decades (Soares, 2016). However, there is no consensus on the effects of taxation (Sarlio-Lähteenkorva & Winkler, 2015). Restrictions on marketing high-fat, sugary and salty food and beverage products to children have had some degree of success (Kraak et al., 2016). According to Swinburn, a political willingness to use policy instruments to drive change will probably be an early hallmark of successful obesity prevention (Swinburn, 2008). In a qualitative study of population level interventions to support family dietary choices, parents called for a reduction in supermarket promotions of unhealthy food and improved access to affordable and high-quality food in local supermarkets. There was a strong message from the parents to policy makers to work with commercial companies to lower the costs of healthy food (Khanom et al., 2015).

In the National Guidance for Primary Care (The Norwegian Directorate of Health, 2010), physicians and PHNs have a special responsibility to prevent, assess and treat overweight and obesity in childhood. The purpose of these guidelines is also to ensure and contribute to a good collaboration across the levels in the health service and across sectors (The Norwegian Directorate of Health, 2010). Simultaneously with efforts on population-based prevention of overweight and obesity in childhood, access to healthcare interventions for weight management should be improved (Abarca-Gómez et al., 2017). Interventions that include diet combined with PA can reduce the risk of obesity in young children (Brown et al., 2019). But what about the emotional and psychological aspects of overweight and obesity? And the structural responsibility? Very few RCT studies include psychological factors in their interventions, which makes for a less than holistic health promotion perspective. Health professionals are poorly prepared for addressing obesity (Dietz et al., 2015) and need guidelines and recommendations for content, perspective and responsibility in relation to promotion and treatment (Nordstrand et al., 2016; Øen & Stormark, 2012). In Norway, the emphasis in the Public Health Act (The Norwegian Ministry of Health and Care Service, 2011) is on all municipal sectors systematically taking health into account to improve population health. Our study shows that the fathers feel uncertain and struggle to understand their responsibility for their child’s obesity, and that this uncertainty and struggle can be seen as an endless combat against factors they have very little opportunity to influence, such as the availability of high-density foods and other structural factors.

The theme *family functioning—negotiating roles and responsibility in parenthood* shows that the fathers feel it is their responsibility and that they do take responsibility, however, they feel uncertain about how to help. The fathers in our study believed it was important to have a good relationship with their children and to follow them up in order to be in a position to help. In contrast, a study by Anti et al. (Anti et al., 2016) found that healthcare providers were of the view that fathers often take a “back seat” instead of a “front seat” when it comes to responsibility in childhood obesity. This was based on their experiences of mostly mothers visiting child health clinics. Holm (Holm, 2008) has argued that a parent has an obligation to give their child a good upbringing within the family context and that this also entails trying to prevent their child becoming obese and intervening if their child does become obese. In a qualitative study (Khandpur et al., 2016), fathers (62%) reported sharing food parenting responsibility with the child’s mother. A study by Mallan et al. (Mallan et al., 2014) found that fathers who reported greater perceived responsibility for feeding their children were more concerned about their child’s weight and had a stronger tendency to control what and how much their child ate. Our study also supports this finding.

The fathers in our study emphasized the importance of recognizing the child’s excess weight and to acknowledge it. These findings are consistent with the findings of Vollmer (Vollmer, 2018), who explored fathers’ perceived role and attempts to prevent childhood obesity in their families. Another study by Júlíusson et al. (Júlíusson et al., 2011) found that parental ability to recognize overweight or underweight in their children was generally poor.

The fathers in our study believed that the family climate affected the child’s eating habits, and a study by Eg et al. (Eg et al., 2017) support this. They found that family interaction was a key factor in how the family handled challenges involved in changing diets and increasing physical activity, and that a family’s internal interactions are important factors for adolescents maintaining weight loss. The families in this study found that challenges in everyday life prevented lifestyle changes being adopted in the family, and that supporting the adolescent was far more difficult and time consuming than expected, and also a cause of family conflicts (Eg et al., 2017). In this same study, it was revealed that divorced parents found it more difficult to agree on what food to serve, and faced great challenges (Eg et al., 2017). The fathers in our study believed that agreement between parents and minimizing conflicts are important for managing lifestyle changes, including when the children live in two-parent households.

Our study has indicated that fathers highlight family functioning as an important factor in childhood weight management and in caring for children and adolescents with overweight or obesity. A literature review by Sigmant-Grant et al. (Sigman-Grant et al., 2015) found that family resilience measures, like family functioning, family structure, routines and family stress, combined with general parenting style, feeding behaviour and parent feeding behaviour, may represent a broader understanding of protective strategies within childhood obesity (Sigman-Grant et al., 2015). This can also relate to negotiations and agreements on tasks, roles and responsibility as our study shows. Resilient families nurture and support each other, face challenges effectively, develop problem-solving skills and have open and direct communication. They are also described as flexible, and have family routines such as shared meals and positive parenting (Coyle, 2011; Luthar et al., 2000). The study by Mazzeschi et al. (Mazzeschi et al., 2013) showed higher levels of dysfunction in parental alliance and family function in families with children with overweight or obesity, meaning that poor parental and family functioning was a predictor of overweight and obesity in adolescence. In a study by East et al. (East et al., 2019), family characteristics that reflect an absence of support for children’s development were associated with overweight and obesity in young adulthood. These findings are in line with the perceptions and experiences of the fathers in our study on the importance of support, collaboration and alliance between the parents.

## 5. Methodological considerations

This study has generated new qualitative knowledge about fathers’ understanding of childhood obesity as well as their parenting role caring for a child with overweight or obesity. This knowledge can be helpful for healthcare professionals and PHNs when they provide help and support to families struggling with overweight or obesity in their children or adolescents. The strength of the study is that it generates knowledge about the father’s perspective by exploring their perceptions and experiences. This paper meets the requirements of the COREQ (Tong et al., 2007) checklist.

Some methodological limitations should be considered when interpreting the results. The self-selection of fathers must be considered. Most of the participating fathers have lived with these problems and challenges related to their children or adolescents for a time. This is a selected group of fathers of children with overweight or obesity who are involved in their children’s development, where the family had contacted the local child health services to obtain help for their children’s weight excess. The fathers exhibited a high degree of self-reflection, and several of them have participated in individual or group consultations with a GP, paediatrician or PHN over a period of time prior to the interviews. This may reflect a selection bias and hence limit transferability of the findings (Lincoln & Guba, 1985). Considering fathers’ extent and quality of involvement and different needs for nutrition education and guidance (Vollmer et al., 2019), areas for future research should include differences by child age and differences by father characteristic, e.g., full time vs part time employment, primary caretaker vs other, in order to tailor guidance and support in counselling.

## 6. Conclusion

Our findings suggest that these fathers perceived their parental role as fathers to be important in childhood weight management. The fathers felt uncertain and struggled to understand their own responsibility for their child’s overweight or obesity. They tried hard to figure out their child’s obesity as a complex interaction of biological, psychosocial, environmental and structural factors. They emphasized the significance of the family functioning, dynamics, roles and responsibility in parenthood. The findings in our study can be used in dialogues between parents and health professionals in clinical practice and child health services with a view to including fathers and helping both fathers and mothers in weight management. It can be necessary to address the significance of family functioning and collaboration within the family. The fathers must be met with understanding about their uncertainty and their struggle to understand their individual responsibility for the child’s weight excess. We believe there is a need for a stronger focus on collective social actions. Addressing both the individual and structural responsibility may reduce the guilt or shame, thus helping these fathers with the weight management of children and adolescents. In order to provide tailored health care, more research is needed to explore the consequences of individual responsibility for fathers and the barriers to managing their child’s overweight or obesity. More research is also needed to explore the conditions for such practices.

## Data Availability

The dataset used and analysed during the current study are available from the corresponding author on reasonable request.

## Abbreviation

GP: General Practitioners
PA: Physical Activity
PHN: Public Health Nurses
